# Historical data as a baseline for conservation: reconstructing long-term faunal extinction dynamics in Late Imperial–modern China

**DOI:** 10.1098/rspb.2015.1299

**Published:** 2015-08-22

**Authors:** Samuel T. Turvey, Jennifer J. Crees, Martina M. I. Di Fonzo

**Affiliations:** 1 Institute of Zoology, Zoological Society of London, Regent's Park, London NW1 4RY, UK; 2 ARC Centre of Excellence for Environmental Decisions, NERP Environmental Decisions Hub, Centre for Biodiversity and Conservation Science, University of Queensland, Brisbane, Queensland 4072, Australia

**Keywords:** dynamic biogeography, evidence-based conservation, range collapse, range fragmentation, gazetteer, gibbon

## Abstract

Extinction events typically represent extended processes of decline that cannot be reconstructed using short-term studies. Long-term archives are necessary to determine past baselines and the extent of human-caused biodiversity change, but the capacity of historical datasets to provide predictive power for conservation must be assessed within a robust analytical framework. Local Chinese gazetteers represent a more than 400-year country-level dataset containing abundant information on past environmental conditions and include extensive records of gibbons, which have a restricted present-day distribution but formerly occurred across much of China. Gibbons show pre-twentieth century range contraction, with significant fragmentation by the mid-eighteenth century and population loss escalating in the late nineteenth century. Isolated gibbon populations persisted for about 40 years before local extinction. Populations persisted for longer at higher elevations, and disappeared earlier from northern and eastern regions, with the biogeography of population loss consistent with the contagion model of range collapse in response to human demographic expansion spreading directionally across China. The long-term Chinese historical record can track extinction events and human interactions with the environment across much longer timescales than are usually addressed in ecology, contributing novel baselines for conservation and an increased understanding of extinction dynamics and species vulnerability or resilience to human pressures.

## 1. Introduction

Understanding the ecological and biogeographic characteristics of population decline is a key area of research in conservation science [1,2]. In particular, accurate information on rates, patterns and drivers of population change under different environmental conditions and human pressures is fundamental for developing appropriate management strategies for threatened species. However, there is continued debate over the existence of general spatial patterns in the dynamic biogeography of extinction events. Range contraction at the scale of a species' range may potentially be determined either by a population's demographic characteristics (the ‘demographic model’, which predicts final persistence near the centre of a species' historical range where populations are larger and less variable) or by the geographical dynamics of threat factors (the ‘contagion model’ or ‘range eclipse’, which predicts final persistence in areas along the edge of a historical range which are impacted last by extinction forces) [3–5]. Similarly, the dynamics of whether species' ranges are likely to fragment as well as contract during population decline, and the expected persistence of small ‘relict’ population isolates at risk from both extrinsic threats and stochastic processes, may be complex and influenced by different ecological conditions and human pressures [6,7]. In order to develop predictive power for conservation management, and understand whether spatial patterns of range decline follow general ecological trends or individualistic, species- or population-specific trajectories, it is necessary to obtain further robust comparative data on real-world case studies of population declines through time.

Extinction events typically represent extended processes of decline in species range and numbers, which may take decades, centuries or even longer to run their course [1,8]. As such, studying remnant modern-day populations of threatened species can often provide only limited information on the dynamics and drivers of the earlier declines that led to their current reduced population status. There is therefore an increasing awareness of the need to integrate historical datasets into conservation research and environmental management, to generate more inclusive decision-making frameworks and provide unique insights into long-term extinction dynamics and the status of both species and ecosystems that are not available from short-term modern ecological studies [9–11]. However, despite recognition of the considerable potential of long-term ecological archives to make important contributions for conservation research, policy and practice, relatively few studies have so far used multi-decadal or longer datasets [11]. In addition, historical data may contain substantial levels of bias and error, associated with processes such as spatially and temporally variable and non-standardized sampling, and data collection by informants lacking scientific training [12–14]. There is therefore an urgent need not only to identify novel historical data sources that can reconstruct past baselines and long-term biodiversity change, but also to assess the usefulness and potential limitations of these data for developing a meaningful understanding of population dynamics through time.

Developing a robust evidence-base on past and present human-caused faunal turnover and extinction is of particular importance for eastern and southeast Asia. This region is experiencing extreme levels of anthropogenic pressure on terrestrial ecosystems, and contains the world's highest proportions of threatened vascular plant, reptile, bird and mammal species [15,16]. All species in some clades biogeographically restricted to eastern and southeast Asia, such as gibbons, are now considered threatened with extinction [17], making research into the vulnerability or resilience of these species to different human pressures an urgent priority. Many Asian ecosystems, notably those in China, have also experienced escalating human overpopulation, natural resource overexploitation and habitat modification for several millennia, and these long-term impacts are likely to have substantially shaped the composition and distribution of regional faunas before the recent historical era [18–20]. Attempts to understand the dynamics and drivers of past regional population losses are therefore of substantial conservation importance. However, there has so far been relatively little attempt to quantify temporal or spatial patterns and environmental correlates of pre-modern biodiversity loss to better understand faunal responses to human pressures in most Asian ecosystems.

China possesses the richest known Late Quaternary palaeontological and zooarchaeological record in the eastern/southeast Asian region [21], and an extensive written historical record going back over two millennia that contains abundant information on past environmental conditions and resources [19,22]. Although pre-modern China lacked a specific ‘scientific’ natural history tradition [23], local gazetteers or *difangzhi* (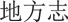) typically recorded considerable local environmental data, including wild animal records, as well as economic, political and demographic information [24]. Compilation of gazetteers at the county level became systematized across China at the beginning of the Ming Dynasty and continued on a regular basis until the mid-twentieth century, with more than 8000 published before 1949, providing dated geographical coverage across most of the country at a reasonably high spatial resolution for the Late Imperial and early modern periods [25,26]. These gazetteers have been used to reconstruct numerous aspects of China's environmental history and its relationship to past changes in Chinese society and economy [27,28]. Published overviews of patterns of early historical elephant, rhino and snub-nosed monkey records across China [19,29,30] and tiger attacks recorded in gazetteers [24,31] suggest that this archive also has the potential to constitute a considerable source of information on the changing historical status of target species of conservation concern, although these data have rarely been investigated within a quantitative analytical framework.

China's current-day mammal fauna includes four surviving gibbon species (eastern hoolock gibbon *Hoolock leuconedys*; black crested gibbon *Nomascus concolor*; Hainan gibbon *N. hainanus*; Cao Vit gibbon *N. nasutus*), all of which are threatened with extinction [17]; the Hainan gibbon is probably the world's rarest living mammal species, with a global population of only 23–25 individuals restricted to a single patch of medium-elevation forest in Bawangling National Nature Reserve, Hainan [32]. Two further gibbon species, the lar gibbon *Hylobates lar* and northern white-cheeked gibbon *Nomascus leucogenys*, have both been extirpated from China during the past couple of decades [33,34]. Like most primates, gibbons are very poorly represented in the Chinese Holocene zooarchaeological record [35]. However, they have represented culturally significant animals for much of Chinese history, often being assigned supernatural or mythic properties, and with their distinctive song symbolizing the melancholy of travellers far from home in traditional literature [36,37]. Their cultural value and morphological distinctiveness led to gibbons being recorded regularly in gazetteers if they were present in the local fauna, in contrast to some other large mammal taxa (e.g. many wild ungulates) that were less readily differentiated by untrained officials [22].

Gazetteer data have previously been used to conduct preliminary investigations of historical extinction patterns and habitat suitability for Chinese gibbons [38,39]. However, these studies have not controlled for issues concerning historical data quality, resolution, incompleteness or biases, or attempted to use information on past extinction dynamics to inform management of currently threatened gibbon populations. In light of the need to assess the quality and usefulness of non-standard data sources for providing novel insights into the status and population dynamics of species of conservation concern, we therefore conducted new analysis of historical gibbon records from China to determine the extent to which the Chinese gazetteer record can be used to reconstruct the dynamic biogeography of extinction events, and whether it can make predictive hypotheses about population vulnerability or resilience that are of direct use in modern conservation.

## 2. Material and methods

### (a) Data

A dataset of 535 dated historical gibbon records from 420 gazetteers (electronic supplementary material, table S1), which provide detailed spatio-temporal coverage for China across the Ming Dynasty (1368–1644), Qing Dynasty (1644–1912) and Republican Period (1912–1949) and with some further sampling of older Jin–Yuan Dynasty records [26], was obtained from a geographical compendium of Chinese gazetteer natural history records [22], constituting a larger dataset compared with previous studies of historical gibbon extinction [39]. This dataset was supplemented with further data on historical (twentieth century and older) and current-day gibbon distributions [17,36,40–42] in order to investigate gibbon population change through time. All Chinese-language records were translated directly by the lead author.

There is considerable potential for error or uncertainty in the identity of animals potentially representing gibbons in old historical records, and historical data were critically assessed and filtered before inclusion. Gibbons are usually differentiated from monkeys in gazetteer records through the use of different names, typically *yuan* (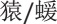) or ‘ape’ for gibbons versus *hou* (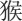) for monkeys [22,36]. However, *yuan* is sometimes locally used to refer to *Trachypithecus* langurs in southern Guangxi [40], and other archaic names sometimes used to refer to gibbons in ancient texts were also possibly used to refer to orang-utans, mythical beings or ethnic minorities [36,43]. In contrast to previous studies [39], records were only accepted as representing gibbons if animals referred to as *yuan* were specifically differentiated from monkeys, if they were referred to using the more descriptive name *changbiyuan* (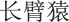, ‘long-armed ape’) and/or if one or more diagnostic features of gibbons (e.g. long arms, good at singing, cannot walk on ground, males and females are different colours) or other relevant characteristics (e.g. arm-bones can be used to make flutes) were also mentioned in the accounts. Records that provided no further information to differentiate the identity of the named animal from a monkey, that included ‘mythic’/‘poetic’ descriptions only or that included inaccurate, conflicting, irrelevant or non-diagnostic descriptions (e.g. an 1873 record of *yuan* from Shangrao, Jiangxi, which refers to the animal's arms but also states that it has a short tail, and otherwise only discusses the animal's kindness and the duration of its pregnancy) were excluded from analysis.

Most gazetteer records do not record specific localities where gibbons occurred, but instead report their presence at the county level [22], making it inappropriate to use precise locality data for spatial analysis [39]. Many county-level boundaries have changed during recent centuries, and so gibbon presence was instead generally recorded at the prefecture level (the administrative level nested hierarchically above county and below province in China), to ensure that historical records were correctly assigned to geographical regions. Spatial data were instead recorded at both district and county/autonomous county levels for Chongqing and at both prefecture and county/autonomous county levels for Hainan, as these administrative regions are geographically non-overlapping rather than nested in these regions, and locally represent the largest sub-province-level geographical divisions. Prefectures and equivalent administrative regions containing gibbon records had a mean ± 1 s.d. area of 13 705 ± 11 145 km^2^ (range: 202–84 110 km^2^). Gibbons from different administrative regions are hereafter referred to as ‘populations’.

The most recent gibbon record for a given administrative unit was interpreted as a last-occurrence date for that region, with gibbons inferred to be regionally present until that date. Gazetteer records of other wild animal species post-dating the latest gibbon records are also reported for most regions, indicating that later regional gibbon absence is unlikely to represent an artefact of incomplete reporting; for example, 82.1% of mainland regions with pre-twentieth century gibbon gazetteer last-occurrence records have younger gazetteer records of tiger, a species known to have survived across much of mainland China until the twentieth century [22]. Nearly all (88.6%) historical gibbon last-occurrence records were associated with an exact calendar year, but a small number were instead only associated with a given date range (e.g. ‘reign of the Qianlong Emperor’ (1735–1796), ‘1950s’). In order to include these data in our analyses, date ranges were converted to direct calendar years by randomly selecting a year from within this range, with an equal probability of being assigned to any year within the range.

### (b) Analysis

Gibbon last-occurrence data were assigned to 50-year time bins from 1600 onwards for most analyses, to permit reconstruction of population dynamics from the Late Imperial period to the present at a level of temporal resolution that accommodated gaps in gazetteer recording (gazetteers were not updated regularly but were updated at least once within a 50-year period; [26]). Older last-occurrence data were used to reconstruct total levels of gibbon spatial distribution across China, but were only used as an initial baseline for comparative analysis due to less systematic gazetteer recording before the Late Imperial period.

We first investigated whether it was possible to detect a switch in the rate of gibbon population extirpation through time as represented by the number of administrative regions occupied by gibbons in each 50-year time bin. We smoothed the time-series using a generalized additive model (GAM; [44]) in order to avoid picking up stochastic fluctuations resulting from environmental variation, and to allow change in mean number of administrative regions to be represented by any smoothed curve shape that best-fits the data [45]. The degree of smoothness of the GAM (controlled by the ‘*k*’ within the model set-up) was constrained to one-third of the time-series length as recommended by Collen *et al*. [46]. We reduced over-fitting of the data by increasing the gamma parameter of the model to the value of 1.4 suggested by Wood [44]. We used a quasi-Poisson error structure to account for the non-normal distribution and overdispersion of our response variable. We detected shifts in time-series dynamics based on switches in the smoothed trend's second derivative sign [2,45–47], which we calculated based on the rate of change of the smoothed trend at each time step. We did this by taking ‘the difference of the difference’ between time steps, and used switches in the second derivative sign (herein termed ‘switch-points') to differentiate between sections of differing dynamics. Negative second derivative sections represented sections where the rate of decline was speeding up, whereas positive second derivative sections represented decreasing rates of decline.

Following the study of Di Fonzo *et al*. [2], we tested that the switch-points were associated with real changes in gibbon population records driven by external pressures and not due to environmental stochasticity by re-calculating switch-points across 100 simulated time-series with similar properties to the focal time-series. We simulated time-series by generating new population records for each year based on the random normal distribution (with the mean equal to the smoothed count for that year and standard deviation equal to 95% CI of smoothed model fit), and defined ‘significant switch-point years' as years that were detected most frequently as switch-points out of all the time-series. In order to relate the second derivative results back to the original data, switch-point years were calculated by adding two time-steps (i.e. two 50-year intervals) to the time-step before the switch in second derivative sign took place. We then determined how the rate of decline was changing over time by fitting linear, quadratic and exponential models to the raw data of each switch-point-delimited section (electronic supplementary material, table S2). We assessed this using a multi-model inference approach [48] based on the model's Akaike's information criterion [49], which we corrected for small sample size (AICc; [50]) to avoid over-fitting. We chose the model with lowest AICc (based on a threshold of Δ*i* > 4; ref. [48]) as best representing the declining trend. The simplest model was selected in cases where the difference in AIC across models was less than 4. If the number of data points within a declining section was two less than the number of parameters within the fitted model, then it was not possible to compute AICc, and we used ΔAIC to compare model fits. If the linear model was best-fit, we re-ran the regressions using a generalized linear modelling (GLM) framework with quasi-Poisson errors to account for non-normality of data.

For each administrative region with gibbon records, the proportion of contiguous neighbouring regions that did not contain gibbons was determined for the overall dataset (corresponding to a time point of ad 250, before any local populations had been extirpated), and for each 50-year interval from 1600 until the last-occurrence date for gibbons from the target region. These proportion data were then averaged across all regions that still contained gibbon populations at each chosen time interval to calculate a gibbon range fragmentation index, which is interpreted as a proxy for population fragmentation and level of isolation or connectivity of gibbon populations. Levels of population fragmentation were considered significantly different between different time intervals if CIs for fragmentation index values did not overlap; 83% CIs were used for comparison because these give an approximate *α* = 0.05 test, whereas comparisons using two sets of 95% CIs are too conservative [51]. The number of gibbon populations that persisted after isolation from populations in all neighbouring regions, and their post-isolation survival time, was also determined.

Finally, we investigated whether the timing of regional gibbon population extinction was associated with any of the following extrinsic environmental or geographical parameters: latitude, longitude, elevation, mean annual precipitation, mean annual temperature, or global human footprint. Regional gibbon last-occurrence dates were converted to years since last sighting and used as the response variable. We also wanted to explore the same relationship using the number of years that gibbon population isolates persisted following complete isolation until local extinction as our response variable, but lacked sufficient data points (*n* = 18) to be able to detect effects with reasonable power [52]. Mean latitude and longitude for all administrative regions containing gibbon records were calculated in the geographic information system (GIS) programme ArcMap [53]. GIS map layers of all climatic and elevation variables were downloaded from the WorldClim database [54] at 30-arc second resolution. We used the Human Footprint Index, a composite measure of current-day human population pressure, land use, infrastructure and access, to approximate anthropogenic impacts across China, downloaded as a GIS map layer from the Last of the Wild database [55]. GIS layers of climatic variables and Human Footprint Index were then overlaid on a map of China in ArcMap, and the average values were aggregated and logged for each administrative region containing gibbon records. We tested for possible collinearity between all variables using variance inflation factors (VIF) in the R package ‘car’; in general, if VIF is found to be above 10, then collinearity is associated with that variable, although this threshold has been debated [56]. All VIFs were found to be less than 5, so all variables were included in the analysis. The relationship between gibbon last-occurrence dates and explanatory variables was modelled using a GLM with a quasi-Poisson error distribution to account for overdispersion [57]. We applied model simplification, deleting variables with the largest *p*-values, and models were checked using the *F*-test to assess subsequent significance of changes in deviance resulting from removal of terms [57]. We also tested for spatial autocorrelation on the final minimum adequate model residuals using Moran's I statistic. All statistical analyses were undertaken using RStudio v. 0.97.551 [58].

## 3. Results

**Figure 1.**
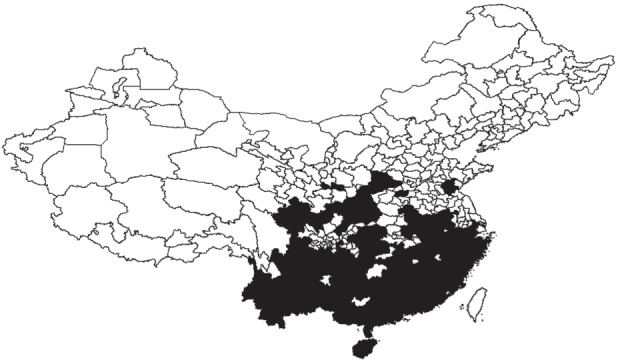
Complete former distribution of gibbons across different administrative regions in China inferred from historical records. Black areas represent regions containing gibbon populations; white areas represent regions with no available records.

Although gibbons are today restricted to 11 prefectures in a small area of southwestern China, we collected gibbon last-occurrence dates ranging from 250 (Fuling, Chongqing) to 1995 (Qiongzhong, Hainan) from a further 149 administrative regions in 19 provinces or equivalent areas distributed across much of central, southern and eastern China (figure 1; electronic supplementary material, table S3).

**Figure 2.**
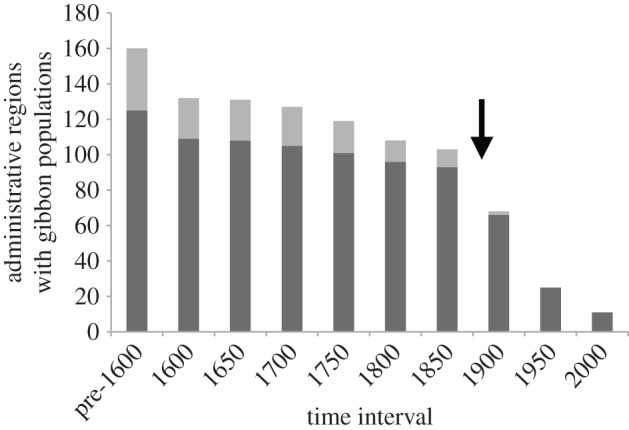
Number of administrative regions containing gibbon populations for complete historical gibbon distribution across China (pre-1600) and over nine consecutive 50-year time intervals (1600–2000). Pale grey, regions north of the Yangtze; dark grey, regions south of the Yangtze. Arrow indicates temporal switch-point in the rate of gibbon population decline.

**Figure 3.**
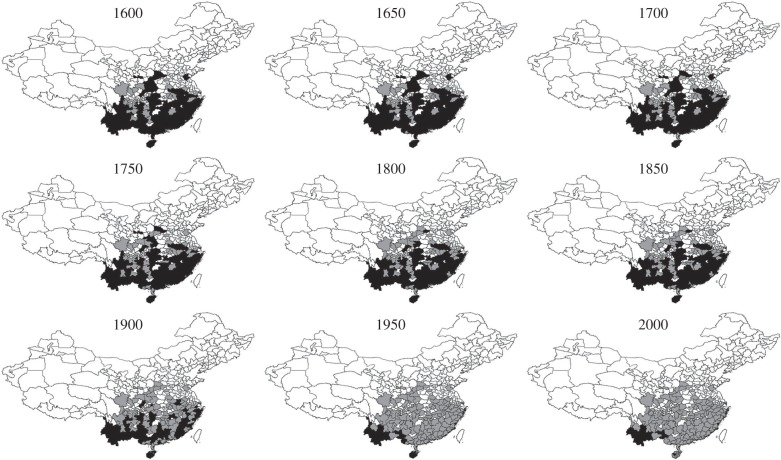
Changing distribution of gibbons across different administrative regions in China over nine consecutive 50-year time intervals (1600–2000). Black areas represent regions containing gibbon populations; grey areas represent regions where gibbons formerly occurred but have been extirpated by a given time interval; white areas represent regions with no available records.

By 1600, gibbons are no longer reported from 17.5% of the regions from which older records are available, and they show a continuous decrease in the number of occupied regions through each successive 50-year intervals (figure 2). We identify a significant switch-point in the rate of this range decrease during the 50-year time-period between 1850 and 1900, which is supported across 100% of our time-series simulations (figure 2). The rate at which gibbon populations were being lost across China escalated significantly after 1850 (GLMs with quasi-Poisson errors: pre-1850, slope = −0.053 ± 0.007 (s.e.), *p* = 0.002; post-1850, slope = −0.698 ± 0.024 (s.e.), *p* = 0.024). We found that linear models best described the time-series sections either side of the switch-point year, suggesting that gibbon populations declined at constant rates over the course of both time intervals (electronic supplementary material, table S4). Only 18.1% of regions stopped reporting gibbons between 1600 and 1850, by which point gibbons are no longer recorded from 36.6% of the regions from which older records are available. By contrast, by 1900 they are no longer recorded from 57.5% of these regions, and by 1950 they are no longer recorded from 84.4% of these regions (figures 2 and 3).

**Figure 4.**
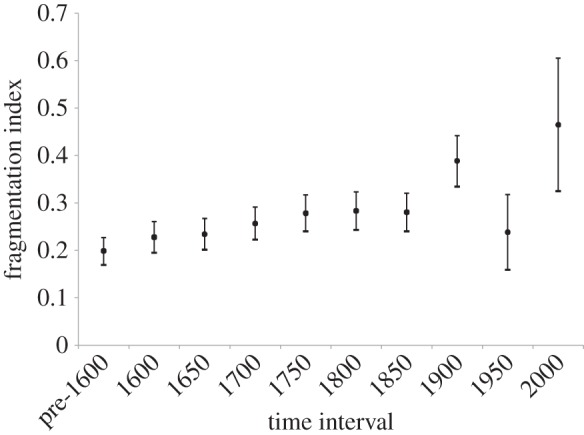
Gibbon fragmentation index and 83% CIs for initial (pre-1600) gibbon distribution across China, and over nine consecutive 50-year time intervals (1600–2000).

The initial fragmentation index value for gibbon populations in our dataset is 0.198 (83% CI: 0.170–0.227), representing the proportion of neighbouring regions that already lack gibbons before any known populations are subsequently lost from the historical record. This starting level of fragmentation in the data may reflect either older human-caused population losses of gibbons, natural environmental heterogeneity meaning that not all neighbouring regions contain suitable natural gibbon habitat within their overall extent of occurrence in China, or spatial gaps in historical reporting; we therefore use this value simply as a relative starting point against which to compare successive fragmentation index values through time. At 1600, the fragmentation index value is 0.228 (83% CI: 0.195–0.260), and fragmentation increases progressively through successive 50-year intervals, until by 1750 it is significantly higher than the starting pre-1600 value (0.278; 83% CI: 0.240–0.317). Fragmentation then increases significantly again between 1850 (0.280; 83% CI: 0.240–0.321) and 1900 (0.388; 83% CI: 0.334–0.442); it then drops significantly by 1950 (0.239; 83% CI: 0.159–0.0.318), and rises again significantly by 2000 (0.465; 83% CI: 0.325–0.605; figure 4).

Nearly all extirpated gibbon populations were last recorded from administrative regions when potentially contiguous populations were still present in neighbouring areas. However, remnant gibbon populations persisted in 18 isolated administrative regions after extirpation of populations that had previously been recorded from all neighbouring areas, and 16 of these isolated populations are also now extinct. Isolated, now-extinct populations were also recorded from a further three regions (Jiangbei, Chongqing; Pingliang, Gansu; Linyi, Shandong) for which no gibbon historical records were available from any neighbouring areas. The mean time to extinction after complete population isolation in the 16 extirpated populations for which last-occurrence data were available for neighbouring areas was 42.9 years, although there was considerable variation around this value (s.d. = 48.8 years, range = 1–172 years).

Although 52.8% of regions south of the Yangtze River still reported gibbons at the start of the twentieth century, the last gibbon record from a region north of the Yangtze (from Dazhou, Sichuan) dates from 1932, and only two regions north of the Yangtze (5.7%) still reported gibbons into the twentieth century (figure 2). Both latitude and longitude were significant predictors of the timing of regional gibbon population extinction (table 1), with gibbons disappearing earlier from more northerly and easterly regions (figure 3). Elevation was also a significant negative predictor of gibbon extinction, with populations persisting for longer at higher elevations. No spatial correlation was found in the final minimum adequate model (Moran I statistic standard deviate = −0.9397, *p* = 0.352).

**Table 1.** Minimum adequate generalized linear model for number of years since local gibbon population extinction in relation to environmental variables. Asterisks denote significance of *p*-values.

	estimate	standard error	*t*-value
intercept	12.159	20.096	3.927
log mean elevation	−0.373	0.092	−4.031***
longitude	−0.079	0.026	−3.029**
latitude	0.155	0.023	6.864***

*p < 0.05, **p < 0.01, ***p < 0.001.

## 4. Discussion

Our investigation of the potential of the long-term Chinese historical record to quantify temporal and spatial dynamics of the extinction process provides important new support that this archive can contribute considerable novel insights for understanding the dynamics of species responses to human pressures, and can track the course of extinction events across much longer timescales than are usually addressed in ecology or conservation biology. Our analyses have controlled or tested for multiple issues affecting data quality, resolution, incompleteness and bias that were not addressed in previous studies, including accurate identification of gibbons from historical records, genuine versus pseudo-absence of gibbons from specific gazetteer archives or geographical regions, analysis within time bins and prefectures to account for spatio-temporal imprecision in original reporting, and spatial autocorrelation. However, it is inevitable that gazetteer data compiled by non-scientific observers cannot provide a complete faunal record at the standard typically expected by modern ecologists. For example, records used in our study are at low taxonomic resolution due to the lack of accompanying morphological detail (electronic supplementary material, table S3), and can be interpreted only as representing generic ‘gibbons' rather than being identifiable to any of the multiple gibbon species known to have occurred historically in China (which remained a source of taxonomic confusion until very recently; [59]). Indeed, it is possible, even likely, that gibbon records from areas of China separated from the ranges of surviving species by major river drainages (e.g. Pearl/Yangtze drainages) that are likely to act as allopatric barriers to gene flow in gibbons [60] may represent undescribed species that became globally extinct during recent centuries. However, the gazetteer record reveals several otherwise unknown aspects of the pattern and process of gibbon population loss across a more than 400-year country-level dataset that cannot be fully understood through consideration of China's surviving remnant gibbon populations. This archive constitutes a particularly useful source of historical data with potential application for conservation, as it provides relatively consistent spatial sampling across the entire geographical area of interest in contrast to other historical archives such as museum collections, which contain substantial levels of spatial reporting bias and omission errors across the distributions of target taxa [13,14].

As suggested by previous studies [39], the spatial pattern of gibbon population decline across China from the Late Imperial period to the present shows strong geographical structuring, with earlier loss of northern and eastern populations and progressive range contraction towards southwestern China (figure 3). This pattern is consistent with the contagion model of range collapse, suggesting that extinction dynamics in Chinese gibbon populations were determined primarily by the pressure of anthropogenic extinction factors that spread directionally across the region, rather than by demographic characteristics of these populations. The observed spatio-temporal pattern of gibbon range eclipse matches known patterns of regional human population density and demographic expansion during the Late Imperial period, with higher initial historical population densities in northern China, Han migration from the north to areas south of the Yangtze from the mid-1500s onwards, and further westward internal expansion from areas of high population density in the southeast, leading to progressive colonization of the southern uplands by Ming and Qing Dynasty settlers (so-called ‘shed people’) [18,31,61]. Gibbon populations therefore appear to have been highly vulnerable to the wavefront of this internal Chinese human population expansion, which would probably have included combined increases in both forest loss and hunting [31].

The contagion model has been proposed as a general pattern for species range collapse [3,4,62], but other studies have found varying support for protracted survival of peripheral subpopulations in a range of species [63–67]. Our results suggest that the contagion model may indeed represent a general biogeographic phenomenon in faunas exposed to major human demographic expansions, and we encourage further reconstruction of historical population changes in other Chinese taxa to assess whether a common faunal response occurred simultaneously across multiple species in this region, or whether different species instead displayed individualistic spatial population trajectories.

Our switch-point analysis demonstrates that gibbon population decline escalated substantially across China from the second half of the nineteenth century onwards (figure 2). This decreasing pattern of gibbon records is highly unlikely to represent a data bias associated with decreased gazetteer reporting, as gazetteer production reached its peak during the Qing Dynasty [26], other natural phenomena (e.g. typhoons) are reported with increased frequency compared to older records during the nineteenth century [24], and other species (e.g. tigers) continue to be reported regularly into the twentieth century [22]. The severe decline in gibbon populations witnessed over the past century is not surprising given the extreme impact on ecosystems across China that resulted from the country's well-documented destructive twentieth-century environmental policies and human population explosion [68]. Escalating twentieth-century environmental pressures also explain the distinctive pattern of a progressive drop and rise in gibbon population fragmentation from 1900 onwards (figure 4), which reflects extirpation of already-fragmented populations and subsequent fragmentation of the last gibbon ‘strongholds’ in far southwestern China and Hainan (figure 3) as anthropogenic pressures on local environments intensified. Indeed, whereas gibbon population loss during recent centuries was indisputably caused by human activities, it is interesting to observe that current-day human pressures on Chinese environments (as measured by the composite Human Footprint Index in our analyses) are unable to predict the dynamics and timing of pre-modern gibbon extinctions, probably because historical spatial variation in regional human impacts across China has been swamped by country-wide intensification of environmental exploitation and destruction over the past century.

However, gibbon population loss escalated before the twentieth century, and although the majority of gibbon range across China was still occupied in 1600, the Late Imperial Era saw progressive population attrition in terms of both geographical area occupied by gibbons and connectivity of gibbon populations, with statistically significant fragmentation apparent by 1750. These quantitative findings are consistent with available contemporary anecdotal historical accounts by European naturalists, which suggest that gibbons were already rare in some parts of China (e.g. Hainan) in the eighteenth and nineteenth centuries [69]. We therefore cannot properly understand gibbon extinction dynamics in China without considering pre-twentieth century regional human interactions with the environment. From an estimated approximately 50% remaining forest cover in 1700, southern China experienced extensive forest clearance throughout the eighteenth century leading to massively reduced regional forest cover by the mid-nineteenth century [31], suggesting that escalating gibbon population extinctions from this point onwards may have represented a pre-modern ‘extinction debt’ in habitats that had already become too degraded to support viable populations in the long-term. Historical records suggest that other mammal species also experienced local population extirpations during the Late Imperial Era (e.g. tigers in Guangzhou prefecture; [31]). Indeed, such historical-era extirpations represent the continuation of a longer-term series of human-caused mammalian losses in China documented across the Holocene, with former native or endemic species such as the short-horned buffalo *Bubalus mephistopheles*, giant muntjac *Muntiacus gigas*, Père David's deer *Elaphurus davidianus*, Asian elephant *Elephas maximus*, Sumatran rhino *Dicerorhinus sumatrensis* and Javan rhino *Rhinoceros sondaicus* all largely or completely extinct across China by the Late Imperial Era [19,21,70,71].

Our analyses of the long-term Chinese gazetteer record not only document the dynamics of past gibbon extinctions, but also provide important historical insights that can inform conservation management of the country's surviving but highly threatened remnant gibbon populations. As previously suggested for gibbons and many other mammal species [29,39,62,72], we demonstrate that gibbon populations occurring at lower elevations in China have been more vulnerable to extinction as a result of greater historical human population growth and habitat conversion in these more accessible regions, and remnant populations are largely restricted to medium/high-elevation montane forests (e.g. eastern hoolock gibbon in Gaoligong Mountains, black crested gibbon in Wuliang Mountains, Hainan gibbon in the Futouling peak region of Bawangling National Nature Reserve; [17,32,42]). Improved understanding of the widespread former occurrence of gibbons in lowland forests across China supports the suggestion that surviving remnant populations may be restricted to suboptimal habitat close to their elevational limit, which has major implications both for understanding the ecological basis of unusual behaviours observed in some of these populations (e.g. unusually large reported home-range and atypical mating system in Hainan gibbons, which may represent responses to low-quality habitat; [73]) and for designing appropriate future management strategies (e.g. spatial planning of forest reconnectivity at Bawangling; [32]). Although considerable variation is seen around the time to extinction of isolated gibbon populations in China across recent centuries, the fact that such populations have a mean survival time of only around 40 years between isolation and extinction provides an important note of urgency for identifying how to manage these surviving populations appropriately. In particular, the only surviving Hainan gibbon population has been completely isolated at extremely low population size since at least 1980 [32,41], making the identification of effective recovery activities for this population an even higher priority.

Our reconstruction of the dynamics and environmental correlates of gibbon population vulnerability and resilience across China represents an important new case study that demonstrates the unique potential of the historical record to understand the extinction process and provide novel baselines for informing conservation. We recommend further investigation of the Chinese gazetteer record to reconstruct long-term human impacts on Chinese ecosystems at a wider faunal level, to determine the chronology of the progressive depletion of the region's fauna and compare responses shown by different species to changing human pressures on local environments throughout recent millennia. We encourage further use of this still-underused resource as a key component of the modern conservation toolkit, that will have to draw upon different complementary types of data in order to prevent future extinctions of highly threatened species in China and elsewhere.

## Supplementary Material

Table S1

## Supplementary Material

Table S2

## Supplementary Material

Table S3

## Supplementary Material

Table S4

## Data accessibility

The datasets supporting this article have been uploaded as part of the electronic supplementary material.
