# Supplementary material for: Historical data as a baseline for conservation: reconstructing long-term faunal extinction dynamics in Late Imperial–modern China
Source: Proc Biol Sci. 2015 Aug 22;282(1813):20151299. doi: 10.1098/rspb.2015.1299 (PMC4632630; doi:10.1098/rspb.2015.1299)
Supplement: Table S1 [file rspb20151299supp1.pdf]

Table S1. List of 420 Chinese gazetteers containing dated gibbon records or other information about gibbons, arranged in chronological order for each province, and showing the number of geographically distinct gibbon records per gazetteer (corresponding to different modern-day Chinese administrative regions). All records accessed and transcribed in the National Library of China by Wen Rongsheng; for further information, see ref [22].

Province	Gazetteer name	Records	Period	Year of publication	Last-occurrence date?
Anhui	*Huizhou Fuzhi*	2	Ming	Hongzhi (1488-1505, date unspecified)	
	Taiping Fuzhi	2	Qing	Kangxi, 12th year (1673)	
	Huizhou Fuzhi	2	Qing	Kangxi, 38th year (1699)	Xuancheng
	Anqing Fuzhi	1	Qing	Kangxi, 60th year (1721)	Anqing
	Taiping Fuzhi	2	Qing	Qianlong, 22nd year (1757)	Ma'anshan
	She Xianzhi	1	Qing	Qianlong, 36th year (1771)	
	Luzhou Fuzhi	3	Qing	Jiaqing, 8th year (1803)	
	Wuwei Zhouzhi	1	Qing	Jiaqing, 8th year (1803)	
	Ningguo Fuzhi	3	Qing	Jiaqing, 20th year (1815)	Wuhu
	Liuan Zhouzhi	1	Qing	Tongzhi, 11th year (1872)	
	Guichi Xianzhi	1	Qing	Guangxu, 9th year (1883)	Chizhou
	Xuxiu Luzhou Fuzhi	3	Qing	Guangxu, 11th year (1885)	Hefei
	Huoshan Xianzhi	1	Qing	Guangxu, 31st year (1905)	Lu'an
	Taiping Xianzhi	1	Qing	Guangxu, 34th year (1908)	Huangshan
	She Xianzhi	1	Republic	26th year (1937)	
Chongqing	*Kuizhou Fuzhi*	1	Qing	Daoguang, 7th year (1827)	
	Chengkou Tingzhi	1	Qing	Daoguang, 24th year (1844)	Chengkou
	Jiangbei Tingzhi	1	Qing	Daoguang, 24th year (1844)	Jiangbei
	Wushan Xianzhi	1	Qing	Guangxu, 19th year (1893)	Wushan
	Nanchuan Xianzhi	1	Republic	15th year (1926)	
	Chongxiu Nanchuan Xianzhi	1	Republic	20th year (1931)	Nanchuan
Fujian	*Sanshan Zhi*	1	Song	Chunxi, 9th year (1182)	
	Bamin Tongzhi Funing Fu	1	Ming	Hongzhi, 4th year (1491)	

	Bamin Tongzhi Fuzhou Fu	2	Ming	Hongzhi, 4th year (1491)	
	Bamin Tongzhi Jianning Fu	2	Ming	Hongzhi, 4th year (1491)	
	Bamin Tongzhi Quanzhou Fu	1	Ming	Hongzhi, 4th year (1491)	
	Bamin Tongzhi Shaowu Fu	2	Ming	Hongzhi, 4th year (1491)	
	Bamin Tongzhi Tingzhou Fu	2	Ming	Hongzhi, 4th year (1491)	
	Bamin Tongzhi Xinghua Fu	1	Ming	Hongzhi, 4th year (1491)	
	Bamin Tongzhi Yanping Fu	2	Ming	Hongzhi, 4th year (1491)	
	Bamin Tongzhi Zhangzhou Fu	1	Ming	Hongzhi, 4th year (1491)	
	Shunchang Yizhi	1	Ming	Zhengde, 17th year (1520)	
	Anxi Xianzhi	1	Ming	Jiajing (1522-1566, date unspecified)	
	Changtai Xianzhi	1	Ming	Jiajing (1522-1566, date unspecified)	
	Funing Zhouzhi	1	Ming	Jiajing (1522-1566, date unspecified)	
	Jangping Fuzhi	1	Ming	Jiajing (1522-1566, date unspecified)	
	Jianning Fuzhi	2	Ming	Jiajing (1522-1566, date unspecified)	
	Jianyang Xianzhi	1	Ming	Jiajing (1522-1566, date unspecified)	
	Longxi Xianzhi	1	Ming	Jiajing (1522-1566, date unspecified)	
	Mingde Xianzhi	1	Ming	Jiajing (1522-1566, date unspecified)	
	Qingliu Xianzhi	1	Ming	Jiajing (1522-1566, date unspecified)	
	Tingzhou Fuzhi	2	Ming	Jiajing (1522-1566, date unspecified)	
	Yanping Fuzhi	2	Ming	Jiajing (1522-1566, date unspecified)	
	Funing Zhouzhi	1	Ming	Wanli, 21st year (1593)	
	Yongan Xianzhi	1	Ming	Wanli, 22nd year (1594)	
	Jianyang Xianzhi	1	Ming	Wanli, 28th year (1600)	
	Fuzhou Fuzhi	2	Ming	Wanli, 41st year (1613)	
	Youyi Xianzhi	1	Ming	Chongzhen, 9th year (1636)	
	Pucheng Xianzhi	1	Qing	Shunzhi, 8th year (1651)	
	Fuqing Xianzhi Xulüe	1	Qing	Kangxi, 6th year (1667)	
	Chongan Xianzhi	1	Qing	Kangxi, 9th year (1670)	
	Dehua Xianzhi	1	Qing	Kangxi, 25th year (1686)	
	Shouning Xianzhi	1	Qing	Kangxi, 25th year (1686)	

	Taining Xianzhi	1	Qing	Kangxi, 25th year (1686)	
	Zhaoan Xianzhi	1	Qing	Kangxi, 30th year (1691)	
	Jianning Fuzhi	2	Qing	Kangxi, 32nd year (1693)	
	Songxi Xianzhi	1	Qing	Kangxi, 39th year (1700)	
	Fuqing Xianzhi	1	Qing	Qianlong, 12th year (1747)	
	Changtai Xianzhi	1	Qing	Qianlong, 13th year (1748)	
	Fuzhou Fuzhi	2	Qing	Qianlong, 16th year (1751)	
	Gutian Xianzhi	1	Qing	Qianlong, 16th year (1751)	
	Tingzhou Fuzhi	2	Qing	Qianlong, 17th year (1752)	
	Yongchun Zhouzhi	1	Qing	Qianlong, 22nd year (1757)	
	Funing Fuzhi	1	Qing	Qianlong, 24th year (1759)	
	Haicheng Xianzhi	1	Qing	Qianlong, 27th year (1762)	
	Longhai Xianzhi	1	Qing	Qianlong, 27th year (1762)	
	Longxi Xianzhi	1	Qing	Qianlong, 27th year (1762)	
	Quanzhou Fuzhi	1	Qing	Qianlong, 28th year (1763)	
	Jiangle Xianzhi	1	Qing	Qianlong, 30th year (1765)	
	Yanping Fuzhi	2	Qing	Qianlong, 30th year (1765)	
	Xianyou Xianzhi	1	Qing	Qianlong, 36th year (1771)	
	Mingde Xianzhi	1	Qing	Qianlong, 46th year (1781)	
	Yongchun Zhouzhi	1	Qing	Qianlong, 52nd year (1787)	Quanzhou
	Zhangping Xianzhi	1	Qing	Daoguang, 10th year (1830)	
	Shunchang Xianzhi	1	Qing	Daoguang, 12th year (1832)	
	Xianyou Xianzhi	1	Qing	Tongzhi, 12th year (1873)	Putian
	Ningyang Xianzhi	1	Qing	Tongzhi, 13th year (1874)	
	Zhangzhou Fuzhi	1	Qing	Guangxu, 3rd year (1877)	
	Funing Fuzhi	1	Qing	Guangxu, 6th year (1880)	Ningde
	Ningyang Xianzhi	1	Qing	Guangxu, 6th year (1880)	
	Chongzuan Shaowu Fuzhi	1	Qing	Guangxu, 24th year (1898)	
	Nanping Xianzhi	1	Republic	8th year (1919)	
	Datian Xianzhi	1	Republic	10th year (1921)	

	Yongtai Xianzhi	1	Republic	11th year (1922)	Fuzhou
	Nan'an Xianzhi	1	Republic	16th year (1927)	
	Yongchun Xianzhi	1	Republic	16th year (1927)	
	Youxi Xianzhi	1	Republic	16th year (1927)	
	Songxi Xianzhi	1	Republic	17th year (1928)	
	Tongan Xianzhi	1	Republic	18th year (1929)	
	Shunchang Xianzhi	1	Republic	25th year (1936)	Nanping
	Liancheng Xianzhi	1	Republic	28th year (1939)	
	Shanghang Xianzhi	1	Republic	28th year (1939)	
	Yongding Xianzhi	1	Republic	30th year (1941)	Longyan
	Zhaoan Xianzhi	1	Republic	31st year (1942)	Zhangzhou
	Putian Xianzhi	1	Republic	34th year (1945)	
	Qingliu Xianzhi	1	Republic	36th year (1947)	Sanming
Gansu	*Pingliang Fuzhi*	1	Ming	Jiajing, 39th year (1560)	
	Huating Xianzhi Wuchan	1	Qing	Jiaqing, 1st year (1796)	Pingliang
Guangdong	*Nanhai Zhi*	7	Yuan	Dade, 8th year (1304)	Shenzhen, Zhongshan, Zhuhai
	Luobushan Zhi	1	Ming	Yongle, 8th year (1410)	
	Deqing Zhouzhi	1	Ming	Jiajing (1522-1566, date unspecified)	
	Guizhou Fuzhi	1	Ming	Jiajing (1522-1566, date unspecified)	
	Huizhou Fuzhi	4	Ming	Jiajing (1522-1566, date unspecified)	
	Nanxiong Zhouzhi	1	Ming	Jiajing (1522-1566, date unspecified)	
	Shixing Xianzhi	1	Ming	Jiajing (1522-1566, date unspecified)	
	Zengcheng Xianzhi	1	Ming	Jiajing (1522-1566, date unspecified)	
	Chaozhou Fuzhi	4	Ming	Jiajing, 26th year (1547)	
	Guangdong Tongzhi Chaozhou Fu	4	Ming	Wanli, 29th year (1601)	
	Guangdong Tongzhi Gaozhou Fu	2	Ming	Wanli, 29th year (1601)	
	Guangdong Tongzhi Huizhou Fu	5	Ming	Wanli, 29th year (1601)	
	Guangdong Tongzhi Leizhou Fu	1	Ming	Wanli, 29th year (1601)	
	Guangdong Tongzhi Nanxiong Fu	1	Ming	Wanli, 29th year (1601)	

	Guangdong Tongzhi Shaozhou Fu	2	Ming	Wanli, 29th year (1601)	
	Guangdong Tongzhi Zhaoqing Fu	5	Ming	Wanli, 29th year (1601)	
	Xingning Xianzhi	1	Ming	Chongzhen, 10th year (1637)	
	Yangshan Xianzhi	1	Qing	Shunzhi, 15th year (1658)	
	Changle Xianzhi	1	Qing	Kangxi, 2nd year (1663)	
	Gaozhou Fuzhi	1	Qing	Kangxi, 11th year (1672)	
	Wuchuan Xianzhi	1	Qing	Kangxi, 11th year (1672)	Zhanjiang
	Buyang Xianzhi	1	Qing	Kangxi, 25th year (1686)	
	Buyuan Xianxinzhi	1	Qing	Kangxi, 25th year (1686)	
	Wengyuan Xianzhi	1	Qing	Kangxi, 25th year (1686)	
	Zengcheng Xianzhi	1	Qing	Kangxi, 25th year (1686)	
	Luodingzhili Zhouzhi	1	Qing	Kangxi, 26th year (1687)	
	Yangchun Xianzhi	2	Qing	Kangxi, 26th year (1687)	
	Yangjiang Xianzhi	1	Qing	Kangxi, 27th year (1688)	
	Xinhui Xianzhi	1	Qing	Kangxi, 29th year (1690)	
	Conghua Xianxinzhi	1	Qing	Kangxi, 49th year (1710)	
	Conghua Xianxinzhi	1	Qing	Yongzheng, 8th year (1730)	
	Lianping Zhouzhi	1	Qing	Yongzheng, 8th year (1730)	
	Baochang Xianzhi	1	Qing	Qianlong, 18th year (1753)	
	Boluo Xianzhi	1	Qing	Qianlong, 28th year (1763)	
	Chaozhou Fuzhi	4	Qing	Qianlong, 40th year (1775)	
	Guishan Xianzhi	1	Qing	Qianlong, 48th year (1783)	
	Zengcheng Xianzhi	1	Qing	Jiaqing, 25th year (1820)	
	Yangjiang Xianzhi	1	Qing	Daoguang, 2nd year (1822)	
	Yongan Xiansanzhi	1	Qing	Daoguang, 2nd year (1822)	
	Dongan Xianzhi	1	Qing	Daoguang, 4th year (1824)	
	Nanxiong Zhouzhi	1	Qing	Daoguang, 4th year (1824)	
	Dianbai Xianzhi	1	Qing	Daoguang, 5th year (1825)	
	Zhaoqing Fuzhi	3	Qing	Daoguang, 13th year (1833)	Foshan
	Huazhou Zhi	1	Qing	Daoguang, 14th year (1834)	

	Changning Xianzhi	1	Qing	Daoguang, 19th year (1839)	
	Fogangxian Zhilijunmin Tingzhi	1	Qing	Daoguang, 22nd year (1842)	
	Changle Xianzhi	1	Qing	Daoguang, 23rd year (1843)	
	Fogangxian Zhilijunmin Tingzhi	1	Qing	Xianfeng, 1st year (1851)	
	Xingning Xianzhi	1	Qing	Xianfeng, 2nd year (1852)	
	Lianzhou Zhi	2	Qing	Tongzhi, 9th year (1870)	
	Wuzhou Fuzhi	1	Qing	Tongzhi, 12th year (1873)	
	Qujiang Xianzhi	1	Qing	Guangxu, 1st year (1875)	
	Huizhou Fuzhi	4	Qing	Guangxu, 3rd year (1877)	Heyuan, Huizhou, Shanwei
	Guangzhou Fuzhi	1	Qing	Guangxu, 5th year (1879)	
	Fangshun Xianzhi	1	Qing	Guangxu, 10th year (1884)	
	Chongxiu Dianbai Xianzhi	1	Qing	Guangxu, 14th year (1888)	
	Gaozhou Fuzhi	1	Qing	Guangxu, 16th year (1890)	Maoming
	Huaxian Zhi	1	Qing	Guangxu, 16th year (1890)	
	Huazhou Xianzhi	1	Qing	Guangxu, 16th year (1890)	
	Chaozhou Fuzhi	4	Qing	Guangxu, 19th year (1893)	
	Xinning Xianzhi	1	Qing	Guangxu, 19th year (1893)	Jiangmen
	Sihui Xianzhi	1	Qing	Guangxu, 22nd year (1896)	Yunfu
	Lianshan Xianzhi	1	Republic	4th year (1915)	
	Huaiji Xianzhi	1	Republic	5th year (1916)	Zhaoqing
	Dongguan Xianzhi	1	Republic	10th year (1921)	Dongguan
	Zengcheng Xianzhi	1	Republic	10th year (1921)	Guangzhou
	Yangjiang Xianzhi	1	Republic	14th year (1925)	
	Shixing Xianzhi	1	Republic	15th year (1926)	Shaoguan
	Chaozhou Fuzhi	4	Republic	22nd year (1933)	Chaozhou, Jieyang, Meizhou, Shantou
	Qingyuan Xianzhi	1	Republic	26th year (1937)	
	Yangshan Xianzhi	1	Republic	27th year (1938)	Qingyuan
	Yangchun Xianzhi	2	Republic	30th year (1941)	Yangjiang
	Xinxiu Dabu Xianzhi	1	Republic	32nd year (1943)	

	Dianbaixian Xinzhigao	1	Republic	35th year (1946)	
Guangxi	*Qinzhou Zhi*	1	Ming	Jiajing (1522-1566, date unspecified)	
	Nanning Fuzhi	4	Ming	Jiajing, 43rd year (1564)	
	Guangxi Taiping Fuzhi	1	Ming	Wanli, 3rd year (1575)	
	Binyang Xianzhi	1	Ming	Wanli, 13th year (1585)	
	Guangdong Tongzhi Lianzhou Fu	3	Ming	Wanli, 29th year (1601)	
	Lianzhou Fuzhi	3	Ming	Chongzhen, 10th year (1637)	Fangchenggang
	Quanzhou Zhi	1	Qing	Kangxi, 28th year (1689)	
	Lianzhou Fuzhi	3	Qing	Kangxi, 60th year (1721)	Beihai
	Lingshan Xianzhi	1	Qing	Yongzheng, 11th year (1733)	Qinzhou
	Wuzhou Fuzhi	1	Qing	Tongzhi, 12th year (1873)	
	Xinning Zhouzhi	1	Qing	Guangxu, 4th year (1878)	
	Pingle Xianzhi	1	Qing	Guangxu, 10th year (1884)	Guilin
	Baise Tingzhi	1	Qing	Guangxu, 17th year (1891)	
	Zhen'an Fuzhi	1	Qing	Guangxu, 18th year (1892)	
	Tengxian Zhi	1	Qing	Guangxu, 34th year (1908)	Wuzhou
	Guiping Xianzhi	1	Republic	9th year (1920)	Guigang
	Luchuan Xianzhi	1	Republic	13th year (1924)	Yulin
	Fuchuan Xianzhi	1	Republic	21st year (1932)	Hezhou
	Tongzheng Xianzhi	1	Republic	22nd year (1933)	
	Shanglin Xianzhi	1	Republic	23rd year (1934)	
	Chongshan Xianzhi	1	Republic	26th year (1937)	
	Yibei Xianzhi	1	Republic	26th year (1937)	Hechi
	Yongning Xianzhi	1	Republic	26th year (1937)	
	Lingyun Xianzhi	1	Republic	31st year (1942)	
	Leiping Xianzhi	1	Republic	35th year (1946)	
	Binyang Xianzhi	1	Republic	37th year (1948)	Nanning
Guizhou	*Guizhou Tongzhi Bijie Wei*	1	Ming	Jiajing (1522-1566, date unspecified)	Bijiecheng
	Guizhou Tongzhi Chengfan Fu	1	Ming	Jiajing (1522-1566, date unspecified)	
	Guizhou Tongzhi Duyun Fu	2	Ming	Jiajing (1522-1566, date unspecified)	

	Guizhou Tongzhi Liping Fu	1	Ming	Jiajing (1522-1566, date unspecified)	
	Guizhou Tongzhi Pu'an Zhou	2	Ming	Jiajing (1522-1566, date unspecified)	Qianxinan
	Guizhou Tongzhi Sinan Fu	1	Ming	Jiajing (1522-1566, date unspecified)	Tongrencheng
	Guizhou Tongzhi Tongren Fu	1	Ming	Jiajing (1522-1566, date unspecified)	
	Guizhou Tongzhi Wusa Wei	1	Ming	Jiajing (1522-1566, date unspecified)	
	Guizhou Tongzhi Yongning Wei	1	Ming	Jiajing (1522-1566, date unspecified)	
	Guizhou Tongzhi Zhenyuan Fu	1	Ming	Jiajing (1522-1566, date unspecified)	Qiandongnan
	Yuqing Xianzhi	1	Qing	Kangxi, 56th year (1771)	
	Xuqian Shu	1	Qing	Qianlong, 44th year (1779)	
	Xuxiu Zheng'an Zhouzhi	1	Qing	Guangxu, 3rd year (1877)	
	Pu'an Zhili Tingzhi	1	Qing	Guangxu, 18th year (1889)	Liupanshui
	Weng'an Xianzhi	1	Republic	3rd year (1913)	Qiannan
	Tongzi Xianzhi	1	Republic	18th year (1929)	
	Xu Zunyi Fuzhi	1	Republic	25th year (1936)	Zunyi
Hainan	*Guangdong Tongzhi Qiongzhou Fu*	1	Ming	Wanli, 29th year (1601)	
	Yanzhou Zhi	1	Ming	Wanli, 41st year (1613)	
	Lehui Xianzhi	1	Qing	Kangxi, 8th year (1669)	
	Qiongshan Xianzhi	1	Qing	Kangxi, 47th year (1708)	
	Lingshui Xianzhi	1	Qing	Qianlong, 57th year (1792)	
	Huitong Xianzhi	1	Qing	Jiaqing, 24th year (1819)	
	Qiongdong Xianzhi	1	Qing	Jiaqing, 25th year (1820)	
	Wanzhou Zhi	1	Qing	Daoguang, 8th year (1828)	
	Qiongzhou Fuzhi	1	Qing	Daoguang, 21st year (1841)	
	Qiongshan Xianzhi	1	Qing	Xianfeng, 7th year (1857)	
	Dingan Xianzhi	1	Qing	Guangxu, 4th year (1878)	Dingan
	Lingao Xianzhi	1	Qing	Guangxu, 18th year (1892)	Lingao
	Yazhou Zhi	1	Qing	Guangxu, 26th year (1900)	
	Lehui Xianzhi	1	Qing	Xuantong, 3rd year (1911)	
	Qiongshan Xianzhi	1	Republic	6th year (1917)	Haikou
	Wenchang Xianzhi	1	Republic	9th year (1920)	Wenchang

	Gan'en Xianzhi	1	Republic	20th year (1931)	
	Hainan Daozhi	1	Republic	22nd year (1933)	
	Yanxian Zhi	1	Republic	25th year (1936)	
Henan	*Guangzhou Zhi*	1	Qing	Guangxu, 13th year (1887)	Xinyang
	Guangshan Xianzhi Yuegao	1	Republic	25th year (1936)	
Hubei	*Huangzhou Fuzhi*	2	Ming	Hongzhi (1488-1505, date unspecified)	Huanggang, Wuhan
	Yiling Zhouzhi	1	Ming	Hongzhi (1488-1505, date unspecified)	
	Badong Xianzhi	1	Ming	Jiajing (1522-1566, date unspecified)	
	Guizhou Quanzhi	1	Ming	Jiajing (1522-1566, date unspecified)	
	Guizhou Zhi	1	Ming	Jiajing (1522-1566, date unspecified)	
	Badong Xianzhi	1	Qing	Kangxi, 22nd year (1683)	
	Huguang Yunyang Fuzhi	1	Qing	Kangxi, 24th year (1685)	
	Hefeng Zhouzhi	1	Qing	Qianlong, 6th year (1741)	
	Donghu Xianzhi	1	Qing	Qianlong, 28th year (1763)	
	Badong Xianzhi	1	Qing	Qianlong, 50th year (1785)	
	Zhushan Xianzhi	1	Qing	Qianlong, 50th year (1785)	
	Hefeng Zhouzhi	1	Qing	Daoguang, 2nd year (1822)	
	Jianshi Xianzhi	1	Qing	Daoguang, 21st year (1841)	
	Xuxiu Donghu Xianzhi	1	Qing	Tongzhi, 3rd year (1864)	
	Fangxian Zhi	1	Qing	Tongzhi, 4th year (1865)	
	Zhushan Xianzhi	1	Qing	Tongzhi, 4th year (1865)	
	Badong Xianzhi	1	Qing	Tongzhi, 5th year (1866)	
	Guizhou Zhi	1	Qing	Tongzhi, 5th year (1866)	
	Jianshi Xianzhi	1	Qing	Tongzhi, 5th year (1866)	
	Laifeng Xianzhi	1	Qing	Tongzhi, 5th year (1866)	
	Yichang Fuzhi	1	Qing	Tongzhi, 5th year (1866)	Yichang
	Zhuxi Xianzhi	1	Qing	Tongzhi, 6th year (1867)	
	Yunyang Xianzhi	1	Qing	Tongzhi, 9th year (1870)	Shiyan
	Badong Xianzhi	1	Qing	Guangxu, 6th year (1880)	Enshi
	Guizhou Zhi	1	Qing	Guangxu, 8th year (1882)	

Hunan	*Yongzhou Fuzhi*	2	Ming	Hongzhi (1488-1505, date unspecified)	
	Yuezhou Fuzhi	3	Ming	Hongzhi (1488-1505, date unspecified)	
	Yongzhou Fuzhi	2	Ming	Zhengde (1506-1521, date unspecified)	
	Changde Fuzhi	1	Ming	Jiajing (1522-1566, date unspecified)	
	Hengzhou Fuzhi	3	Ming	Jiajing (1522-1566, date unspecified)	
	Yuezhou Fuzhi	3	Ming	Longqing (1567-1572, date unspecified)	
	Chenzhou Zhi	1	Ming	Wanli (1573-1619, date unspecified)	
	Lingling Xianzhi	1	Qing	Kangxi, 23rd year (1684)	
	Changsha Fuzhi	5	Qing	Kangxi, 24th year (1685)	
	Chenzhou Zhi	1	Qing	Kangxi, 24th year (1685)	
	Yongding Weizhi	1	Qing	Kangxi, 24th year (1685)	
	Lingling Xianzhi	1	Qing	Kangxi, 48th year (1709)	
	Qianyang Xianzhi	1	Qing	Yongzheng, 11th year (1733)	
	Yuezhou Fuzhi	1	Qing	Qianlong, 11th year (1746)	
	Changsha Fuzhi	5	Qing	Qianlong, 12th year (1747)	Changsha
	Chunan Miaojiang	1	Qing	Qianlong, 15th year (1750)	
	Zhikang Lizhou Zhilin	1	Qing	Qianlong, 15th year (1750)	
	Pingjiang Xianzhi	1	Qing	Qianlong, 20th year (1755)	
	Xiangtan Xianzhi	1	Qing	Qianlong, 21st year (1756)	Xiangtan
	Yongqing Xianzhi	1	Qing	Qianlong, 27th year (1762)	
	Hengzhou Fuzhi	2	Qing	Qianlong, 28th year (1763)	Zhuzhou
	Chengzhou Fuzhi	1	Qing	Qianlong, 30th year (1765)	
	Yongshun Xianzhi	1	Qing	Qianlong, 58th year (1793)	
	Guiyang Xianzhi	1	Qing	Jiaqing, 7th year (1802)	
	Yizhang Xianzhi	1	Qing	Jiaqing, 20th year (1815)	
	Linwu Xianzhi	1	Qing	Jiaqing, 22nd year (1817)	
	Longshan Xianzhi	1	Qing	Jiaqing, 23rd year (1818)	
	Yongding Xianzhi	1	Qing	Daoguang, 3rd year (1823)	
	Yongzhou Fuzhi	2	Qing	Daoguang, 8th year (1828)	
	Xinhua Xianzhi	1	Qing	Daoguang, 12th year (1832)	Loudi

	Guidong Xianzhi	1	Qing	Xianfeng, 9th year (1859)	
	Guidong Xianzhi	1	Qing	Tongzhi, 5th year (1866)	
	Guiyang Xianzhi	1	Qing	Tongzhi, 6th year (1867)	
	Lizhou Zhi	1	Qing	Tongzhi, 8th year (1869)	Zhangjiajie
	Xuxiu Yongding Xianzhi	1	Qing	Tongzhi, 8th year (1869)	
	Jianghua Xianzhi	1	Qing	Tongzhi, 9th year (1870)	
	Baojing Xianzhi	1	Qing	Tongzhi, 10th year (1871)	
	Pingjiang Xianzhi	1	Qing	Tongzhi, 13th year (1874)	Yueyang
	Hengshan Xianzhi	1	Qing	Guangxu, 1st year (1875)	Hengyang
	Xiangyin Xiantuzhi	1	Qing	Guangxu, 6th year (1880)	
	Yongxing Xianzhi	1	Qing	Guangxu, 9th year (1883)	Chenzhou
	Taoyuan Xianzhi	1	Qing	Guangxu, 18th year (1892)	Changde
	Yongming Xianzhi	1	Qing	Guangxu, 33rd year (1901)	Yongzhou
	Yuanling Xianzhi	1	Qing	Guangxu, 34th year (1902)	Huaihua
	Yongshun Xianzhi	1	Republic	19th year (1930)	Xiangxi
	Dao Xianzhi	1	Republic	20th year (1931)	
Jiangsu	*Wudu Fu*	1	Jin	265-420 (date unspecified)	Suzhou
Jiangxi	*Huizhou Fuzhi*	1	Ming	Hongzhi (1487-1505, date unspecified)	
	Xincheng Xianzhi	1	Ming	Zhengde (1506-1521, date unspecified)	
	Yuanzhou Fuzhi	2	Ming	Zhengde (1506-1521, date unspecified)	
	Ganzhou Fuzhi	1	Ming	Jiajing (1522-1566, date unspecified)	
	Guangxin Fuzhi	1	Ming	Jiajing (1522-1566, date unspecified)	
	Jiujiang Fuzhi	1	Ming	Jiajing (1522-1566, date unspecified)	
	Nan'an Fuzhi	1	Ming	Jiajing (1522-1566, date unspecified)	
	Ningzhou Zhi	1	Ming	Jiajing (1522-1566, date unspecified)	
	Yuanzhou Fuzhi	2	Ming	Jiajing (1522-1566, date unspecified)	
	Xinxiu Nanchang Fuzhi	2	Ming	Wanli, 15th year (1587)	
	Guangxin Junzhi	1	Qing	Kangxi, 22nd year (1683)	
	Ruijian Xianzhi	1	Qing	Kangxi, 22nd year (1683)	
	Wanzai Xianzhi	1	Qing	Kangxi, 22nd year (1683)	

	Huizhou Fuzhi	1	Qing	Kangxi, 38th year (1699)	
	Jiangxi Tongzhi	1	Qing	Yongzheng, 10th year (1732)	
	Jiangxi Xincheng Xianzhi	1	Qing	Qianlong, 15th year (1750)	
	Yuanzhou Fuzhi	2	Qing	Xianfeng, 10th year (1860)	Pingxiang
	Leping Xianzhi	1	Qing	Tongzhi, 9th year (1870)	Jingdezhen
	Wuning Xianzhi	1	Qing	Tongzhi, 9th year (1870)	Jiujiang
	Jiangxi Xincheng Xianzhi	1	Qing	Tongzhi, 10th year (1871)	Fuzhou
	Guangfang Xianzhi	1	Qing	Tongzhi, 11th year (1872)	Shangrao
	Ganzhou Fuzhi	1	Qing	Tongzhi, 12th year (1873)	Ganzhou
	Nanchang Fuzhi	2	Qing	Tongzhi, 12th year (1873)	Nanchang
	Qianshan Xianzhi	1	Qing	Tongzhi, 12th year (1873)	
	Nan'an Fuzhi Buzheng	1	Qing	Guangxu, 1st year (1875)	
	Yichun Xianzhi	1	Republic	29th year (1940)	Yichun
Shaanxi	*Lüeyang Xianzhi*	1	Ming	Jiajing (1522-1566, date unspecified)	Hanzhong
	Shaanxi Tongzhi	1	Ming	Jiajing, 26th year (1547)	
	Xunyang Xianzhi	1	Qing	Yongzheng, 9th year (1731)	Ankang
	Shaanxi Tongzhi Shanzhou	1	Qing	Yongzheng, 13th year (1735)	
	Shaanxi Tongzhi Huayue	1	Qing	Yongzheng, 13th year (1735)	Weinan
	Shangzhou Zhi	1	Qing	Qianlong (1735-1796, date unspecified)	Shangluo
	Xian Fuzhi	1	Qing	Qianlong, 44th year (1779)	Xi'an
Shandong	*Shandong Tongzhi*	1	Qing	Qianlong, 1st year (1736)	Linyi
Shanxi	*Jiangxian Zhi*	1	Qing	Shunzhi, 16th year (1659)	Yuncheng
	Zhaocheng Xianzhi	1	Qing	Qianlong, 25th year (1760)	Linfen
	Yangcheng Xianzhi	1	Qing	Tongzhi, 13th year (1874)	Jincheng
Sichuan	*Hongya Xianzhi*	1	Ming	Jiajing (1522-1566, date unspecified)	Meishan
	Mahu Fuzhi	3	Ming	Jiajing (1522-1566, date unspecified)	Leshan, Yibin
	Qingxi Xianzhi	1	Qing	Jiaqing, 4th year (1799)	Ya'an
	Qiongxi Yelu	2	Qing	Xianfeng, 9th year (1859)	Panzhihua
	Huili Zhouzhi	1	Qing	Tongzhi, 13th year (1874)	
	Leibo Tingzhi	1	Qing	Guangxu, 19th year (1893)	Liangshan

	Mingshan Xianxinzhi	1	Republic	19th year (1930)	
	Xuanhan Xianzhi	1	Republic	20th year (1931)	
	Wanyuan Xianzhi	1	Republic	21st year (1932)	Dazhou
	Guanxian Zhi	1	Republic	22nd year (1933)	
	Hanyuan Xianzhi	1	Republic	30th year (1940)	
Yunnan	*Yunnan Zhi Guangxi Fu*	3	Ming	Zhengde (1506-1521, date unspecified)	
	Yunnan Fuzhi	1	Qing	Kangxi, 35th year (1696)	Kunming
	Jianshui Xianzhi	1	Qing	Yongzheng, 9th year (1731)	
	Guangxi Fu	3	Qing	Qianlong, 4th year (1739)	
	Yunnan Tongzhi Dianxi	1	Qing	Jiaqing, 13th year (1808)	
	Zhaozhou Zhi	1	Qing	Daoguang, 18th year (1838)	
	Xuanwei Zhouzhi	1	Qing	Daoguang, 24th year (1844)	
	Nanning Xianzhi	1	Qing	Xianfeng, 2nd year (1852)	
	Yongchang Fuzhi	1	Qing	Guangxu, 11th year (1885)	
	Zhanyi Zhouzhi	1	Qing	Guangxu, 11th year (1885)	
	Yunnan Xianzhi	1	Qing	Guangxu, 16th year (1890)	
	Zhennan Zhouzhilüe	1	Qing	Guangxu, 18th year (1982)	
	Langqiong Xianzhilüe	1	Qing	Guangxu, 28th year (1902)	Dali
	Shunning Fuzhi	6	Qing	Guangxu, 30th year (1904)	Qujing
	Longling Xianzhi	1	Republic	6th year (1917)	
	Yuanjiang Zhigao	1	Republic	11th year (1922)	
	Maguan Xianzhi	1	Republic	21st year (1932)	Wenshan
	Xuanwei Xianzhigao	1	Republic	23rd year (1934)	
	Tengchong Xianzhigao	1	Republic	30th year (1941)	
	Qiaojia Xianzhi	1	Republic	31st year (1942)	Zhaotong
	Yaoan Xianzhi	1	Republic	37th year (1948)	
Zhejiang	*Shan Lu*	1	Song	Jiading, 7th year (1214)	
	Chicheng Zhi	1	Song	Jiading, 16th year (1223)	
	Yueqing Xianzhi	1	Ming	Yongle (1403-1424, date unspecified)	
	Anji Zhouzhi	1	Ming	Jiajing (1522-1566, date unspecified)	

	Taiping Xianzhi	1	Ming	Jiajing (1522-1566, date unspecified)	
	Zhejiang Tongzhi	1	Ming	Jiajing (1522-1566, date unspecified)	
	Wuyi Xianzhi	1	Ming	Jiajing, 2nd year (1523)	
	Kuaiji Xianzhi	1	Ming	Wanli (1573-1619, date unspecified)	
	Qiantang Xianzhi	1	Ming	Wanli, 37th year (1609)	
	Longquan Xianzhi	1	Qing	Shunzhi, 12th year (1655)	
	Jinyun Xianzhi	1	Qing	Kangxi, 10th year (1671)	
	Shengxian Zhi	1	Qing	Kangxi, 10th year (1671)	
	Jiangshan Xianzhi	1	Qing	Kangxi, 40th year (1701)	
	Quzhou Fuzhi	1	Qing	Kangxi, 50th year (1711)	
	Qiantang Xianzhi	1	Qing	Kangxi, 57th year (1718)	
	Anji Zhouzhi	1	Qing	Qianlong, 15th year (1750)	Huzhou
	Xuanping Xianzhi	1	Qing	Qianlong, 18th year (1753)	Jinhua
	Shaoxing Fuzhi	2	Qing	Qianlong, 57th year (1792)	
	Taishun Fenjiang Lu	1	Qing	Tongzhi, 4th year (1865)	
	Shengxian Zhi	1	Qing	Tongzhi, 9th year (1870)	
	Jiangshan Xianzhi	1	Qing	Tongzhi, 12th year (1873)	
	Jinyun Xianzhi	1	Qing	Guangxu, 2nd year (1876)	
	Qingyuan Xianzhi	1	Qing	Guangxu, 3rd year (1877)	
	Fenjiang Lu	1	Qing	Guangxu, 4th year (1878)	
	Longquan Xianzhi	1	Qing	Guangxu, 4th year (1878)	
	Zhenhai Xianzhi	1	Qing	Guangxu, 5th year (1879)	
	Quzhou Fuzhi	1	Qing	Guangxu, 8th year (1882)	
	Cixi Xianzhi	1	Qing	Guangxu, 14th year (1888)	
	Shangyu Xianzhi	1	Qing	Guangxu, 17th year (1891)	
	Xianju Zhi	1	Qing	Guangxu, 20th year (1894)	
	Suichang Xianzhi	1	Qing	Guangxu, 22nd year (1896)	Lishui
	Yuqian Xianzhi	1	Qing	Guangxu, 24th year (1898)	
	Cixi Xianzhi	1	Qing	Guangxu, 25th year (1899)	
	Lin'an Xianzhi	1	Qing	Xuantong, 2nd year (1910)	

	Yueqing Xianzhi	1	Republic	1st year (1912)	Wenzhou
	Shengxian Zhi	1	Republic	7th year (1918)	
	Jiande Xianzhi	1	Republic	8th year (1919)	
	Hangzhou Fuzhi	2	Republic	11th year (1922)	Jiaxing
	Quxian Zhi	1	Republic	15th year (1926)	Quzhou
	Taizhou Fuzhi	2	Republic	15th year (1926)	Ningbo
	Xiangshan Fuzhi	1	Republic	16th year (1927)	
	Linhai Xianzhi	1	Republic	23rd year (1934)	Taizhou
	Kuaiji Xianzhigao	1	Republic	25th year (1936)	Shaoxing
