# Supplementary material for: Historical data as a baseline for conservation: reconstructing long-term faunal extinction dynamics in Late Imperial–modern China
Source: Proc Biol Sci. 2015 Aug 22;282(1813):20151299. doi: 10.1098/rspb.2015.1299 (PMC4632630; doi:10.1098/rspb.2015.1299)
Supplement: Table S2 [file rspb20151299supp2.pdf]

Table S2. Model equations describing different decline-curve shapes.

Model	Formula	Parameters
Linear	$N_i = m_1 \mathrm{T_i} + c$	N_i represents population size at time i, T_i represents year i, c represents the model intercept at T_{we} = 0, m_1 represents the model slope.
Quadratic	$N_i = m_1 \mathrm{T_i} + m_2 \mathrm{T_i}^2 + c$	N_i represents population size at time i, T_i represents year i, c represents the model intercept at T_{we} = 0, m_1 *and* m_2 represent different model slopes.
Exponential	$N_i = Asym + (RO - Asym)\mathrm{e}^{-\mathrm{lrcT_i}}$	N_i represents population size at time i, *Asym* represents the horizontal asymptote of the model, *RO* represents the intercept at T_i = 0, *lrc* represents the model constant (i.e. decay rate).
