# Supplementary material for: Historical data as a baseline for conservation: reconstructing long-term faunal extinction dynamics in Late Imperial–modern China
Source: Proc Biol Sci. 2015 Aug 22;282(1813):20151299. doi: 10.1098/rspb.2015.1299 (PMC4632630; doi:10.1098/rspb.2015.1299)
Supplement: Table S3 [file rspb20151299supp3.pdf]

**Table S3.** Last-occurrence records for gibbons across different administrative regions in China.

| Province | Administrative region | N or S of Yangtze | Last record | Randomised date | Ref. | Description and notes |
|---|---|---|---|---|---|---|
| Anhui | Anqing | N | 1721 | | 1 | "yuan (like monkey but bigger, with long arms)" |
| Anhui | Chizhou | S | 1883 | | 1 | "yuan, hou" found at Gongchi |
| Anhui | Chuzhou | N | 762 | | 2 | at Taiping |
| Anhui | Hefei (incl. Chaohu) | N | 1885 | | 1 | "changbiyuan" |
| Anhui | Huangshan | S | 1908 | | 1 | "yuan, hou"; gibbons also specifically referred to in 1815 record; also several other local records, but mostly inaccurate/mythological |
| Anhui | Lu'an | N | 1905 | | 1 | "yuan, hou"; gibbons also specifically referred to in 1803 and 1885 records |
| Anhui | Ma'anshan | N | 1757 | | 1 | "good at singing, best at moving" |
| Anhui | Wuhu | S | 1815 | | 1 | "gibbons" in Nanling |
| Anhui | Xuancheng | S | 1699 | | 1 | "changbiyuan" in Jixi; more recent record from 1815 provides only poetic description of gibbons, so may represent vague memory of past gibbon occurrence rather than contemporary survival |
| Chongqing | Chengkou | N | 1844 | | 1 | "yuan (loudly calling/shouting yellow monkey, long arms, good at singing, good at climbing), monkeys, smaller type of monkeys" |
| Chongqing | Fengjie | S | 762 | | 2 | at Kuizhou |
| Chongqing | Fuling | S | 250 | | 2 | |
| Chongqing | Jiangbei | N | 1844 | | 1 | "yuan at Tonglingjing (has golden hair, long arms, and long fur)" - not very detailed description but does mention long arms so potentially accurate |
| Chongqing | Nanchuan | S | 1931 | | 1 | "yuan" (classified in the monkey group … like a monkey but bigger, long arms, good at channelling qi energy, lives for a very long time … arm bones can be used to make flutes … different coloured types (clear-coloured, white, black, yellow, red) … calm/peaceful nature ... sings a call composed of three notes); also similar description from 1926 |
| Chongqing | Shizhu Tujia | S | 11th century | 1015 | 2 | at Wanshou Mountain |
| Chongqing | Wushan | N | 1893 | | 1 | "hou, yuan"; yuan described with reference to classical poetry and with description |

| | | | | | | |
|---|---|---|---|---|---|---|
| | | | | | | of yellow and black types, like monkey but larger, long arms |
| Fujian | Fuzhou | S | 1922 | | 1 | "yuan - like monkey but with long arms and a clear call", found in Yongtai County |
| Fujian | Longyan | S | 1941 | | 1 | "hou, yuan (like monkey, long arms, good at singing)" |
| Fujian | Nanping | S | 1936 | | 1 | "yuan - like monkey but with long arms and a clear call, and good at climbing", found at Shunchang; several other early 20th century and many older records |
| Fujian | Ningde | S | 1880 | | 1 | "hou, yuan, xingxing" |
| Fujian | Putian | S | 1873 | | 1 | "yuan (distinct from monkey, has long arms, good at singing)"; 1945 record reports "two types of hou (small one (yuan), and large one (xingxing) which is rarely seen), so gibbons probably already locally extinct by this date |
| Fujian | Quanzhou | S | 1787 | | 1 | "yuan, hou"; 1927 record states that "yuan" are seldom seen, so possibly already locally extinct by this date |
| Fujian | Sanming | S | 1947 | | 1 | |
| Fujian | Zhangzhou | S | 1942 | | 1 | |
| Gansu | Pingliang | N | 1796 | | 1 | "yuan, hou" found at Huating |
| Guangdong | Chaozhou | S | 1933 | | 1 | "hou, yuan" |
| Guangdong | Dongguan | S | 1921 | | 1 | "yuan, hou"; records from 1879 and 1911 explicitly mention "changbiyuan" |
| Guangdong | Foshan | S | 1833 | | 1 | "changbiyuan" |
| Guangdong | Guangzhou | S | 1921 | | 1 | "hou, yuan"; similar records from 1890 and 1911, naming monkeys and apes separately and providing description of gibbon ("long arms, and with yellow, black and white types") |
| Guangdong | Heyuan | S | 1877 | | 1 | "changbiyuan" |
| Guangdong | Huizhou | S | 1877 | | 1 | "yuan, hou" |
| Guangdong | Jiangmen | S | 1893 | | 1 | "hou, yuan"; record from 1941 states that gibbon and other species are now regionally extinct |
| Guangdong | Jieyang | S | 1933 | | 1 | "changbiyuan" |
| Guangdong | Maoming | S | 1890 | | 1 | "ape, stone monkey ["shihou" – possibly karst-dwelling langur?], monkey"; amongst descriptions of primates in this account, mentions an animal that can sing well and specifically states that females are yellow and males are black; however, another 1890 report from the same region states that both monkeys and apes are no longer regionally present; previous verifiable report is from 1888, which mentions "yuan, hou" and singing ability of the ape; record from 1946 does not list apes |

| | | | | | | |
|---|---|---|---|---|---|---|
| Guangdong | Meizhou | S | 1933 | | 1 | "changbiyuan"; other record of "changbiyuan" from 1893; record from 1943 only records monkeys |
| Guangdong | Qingyuan | S | 1938 | | 1 | "yuan, hou: monkey has noisy temperament, ape has calm temperament and long arms"; other good description from 1937 (describing sorrowful quality of calls); additional record from 1915 ("yuan, hou") |
| Guangdong | Shantou | S | 1933 | | 1 | "changbiyuan" |
| Guangdong | Shanwei | S | 1877 | | 1 | "changbiyuan" |
| Guangdong | Shaoguan | S | 1926 | | 1 | "yuan, hou"; record from 1839 provides accurate description of gibbon ("like monkey but larger, long arms", etc) |
| Guangdong | Shenzhen | S | 1304 | | 1 | "changbiyuan" |
| Guangdong | Yangjiang | S | 1941 | | 1 | "yuan, macaque", with lengthy description of gibbon; record from 1925 reports "yuan, hou" with gibbon description |
| Guangdong | Yunfu | S | 1896 | | 1 | "ape (has yellow, black and white types [male and female?])" |
| Guangdong | Zhanjiang | S | 1672 | | 1 | "ape (long arms, yellow fur, two types [male and female?])" |
| Guangdong | Zhaoqing | S | 1916 | | 1 | "monkey, white ape, black ape [male and female?]"; record from 1873 explicitly names "changbiyuan" |
| Guangdong | Zhongshan | S | 1304 | | 1 | "changbiyuan" |
| Guangdong | Zhuhai | S | 1304 | | 1 | "changbiyuan" |
| Guangxi | Baise | S | extant | | 6 | |
| Guangxi | Beihai | S | 1721 | | 1 | "has yuan, has hou" |
| Guangxi | Chongzuo | S | 1950s | 1958 | 3 | |
| Guangxi | Fangchenggang | S | 1637 | | 1 | "changbiyuan" |
| Guangxi | Guigang | S | 1920 | | 1 | "hou, yuan" (together with description of the respective characters of these two primates - not biologically very useful, but emphasis on honest/merciful characters of gibbon in contrast to greedy/nasty character of monkey) |
| Guangxi | Guilin | S | 1884 | | 1 | "yuan, hou" |
| Guangxi | Hechi | S | 1937 | | 1 | "yuan, hou" |
| Guangxi | Hezhou | S | 1932 | | 1 | "yuan" - mentions apes in a long list of animal types |
| Guangxi | Liuzhou | S | 805-819 | 818 | 2 | |
| Guangxi | Nanning | S | 1948 | | 1 | "yuan, hou"; record from 1937 reports "black apes, macaques", with description of gibbon (long arms, etc) |

| | | | | | | |
|---|---|---|---|---|---|---|
| Guangxi | Qinzhou | S | 1733 | | 1 | "yuan, hou" |
| Guangxi | Wuzhou | S | 1908 | | 1 | "xingxing, yuanhou / yuan, hou" |
| Guangxi | Yulin | S | 1924 | | 1 | "hou, yuan" (together with description of the respective characters of these two primates, similar to description associated with 1924 date at Guigang) |
| Guizhou | Bijiecheng | S | 1522-1566 | 1545 | 1 | "changbiyuan" |
| Guizhou | Guiyang | S | 1741 | | 2 | |
| Guizhou | Liupanshui | S | 1889 | | 1 | "has hou, has yuan" |
| Guizhou | Qiandongnan | S | 1522-1566 | 1541 | 1 | "changbiyuan" |
| Guizhou | Qiannan | S | 1913 | | 1 | "yuan, hou" |
| Guizhou | Qianxinan | S | 1522-1566 | 1550 | 1 | "changbiyuan" |
| Guizhou | Tongrencheng | S | 1522-1566 | 1532 | 1 | "yuan, hou" |
| Guizhou | Zunyi | S | 1936 | | 1 | simply records that apes are found in the eastern edge of the county, but 1929 report provides more detailed description that identifies the animals as gibbons (like monkey but larger, black in colour) |
| Hainan | Baisha | S | extant | | 6 | |
| Hainan | Baoting | S | 1983 | | 4 | |
| Hainan | Changjiang | S | extant | | 6 | |
| Hainan | Chengmai | S | 1964 | | 4 | |
| Hainan | Danzhou | S | 1950 | | 4 | |
| Hainan | Dingan | S | 1878 | | 1 | "yuan, hou" (three types of ape - golden-furred, clear faced, and black - clear-faced is smaller) |
| Hainan | Dongfang | S | 1978 | | 4 | |
| Hainan | Haikou (=Qiongshan) | S | 1917 | | 1 | "yuan", with detailed description that matches gibbon (like monkey but bigger, long arms, etc) |
| Hainan | Ledong | S | 1983 | | 4 | |
| Hainan | Lingao | S | 1892 | | 1 | "yuan, hou" |
| Hainan | Lingshui | S | 1983 | | 4 | |
| Hainan | Qionghai | S | 1964 | | 4 | |
| Hainan | Qiongzhong | S | 1995 | | 4 | |
| Hainan | Sanya | S | 1964 | | 4 | |
| Hainan | Tunchang | S | 1964 | | 4 | |

| | | | | | | |
|---|---|---|---|---|---|---|
| Hainan | Wanning | S | 1983 | | 4 | |
| Hainan | Wenchang | S | 1920 | | 1 | refers to "monkeys", but apparently using this word in equivalent sense to "primate", as also talks about long arms, black fur, etc. |
| Hainan | Wuzhishan | S | 1983 | | 4 | |
| Henan | Xinyang | N | 1887 | | 1 | Huangchuan County has "yuan: ... like monkey but bigger, extremely long arms that can channel qi energy, clear [qing], white, dark, yellow and red types, quiet and still nature, eats fruit..."; 1936 record reports that the region used to have numerous species, including "every type of yuan", but these have all now vanished |
| Henan | Zhengzhou | N | 762 | | 2 | at Huangchuan |
| Henan | Zhumadian | N | 416 | | 2 | at Dengfeng, Songshan |
| Hubei | Enshi Tujia and Miao Autonomous Prefecture | S | 1880 | | 1 | "yuan, hou"; one 1866 record reports that monkeys are commonly called macaque, but the large ones are called yuan, whereas another 1866 record reports that "yuanhou" is "now rare", and an 1841 record lists them as dangerous to people (along with muntjacs etc), again suggesting that knowledge about them is being lost; Van Gulik [ref. 2] also contains good description of gibbons from 1827 |
| Hubei | Huanggang | N | 1488-1505 | 1490 | 1 | "yuan, hou" |
| Hubei | Shiyan | N | 1870 | | 1 | "yuan, hou"; other similar records from 1685, 1785, 1865 (twice) and 1867 |
| Hubei | Wuhan | N | 1488-1505 | 1491 | 1 | "yuan, hou" |
| Hubei | Xiaogan | N | 762 | | 2 | at Anlu |
| Hubei | Yichang | N | 1866 | | 1 | "yuan" and "solitary-ape" (duyuan); similar record from 1864, and also from 1763 and from Ming Dynasty (1488-1505, 1522-1566) |
| Hunan | Changde | S | 1892 | | 1 | "like macaque but bigger and with more quiet character ... has long arms, good at singing and climbing, etc ... also explicitly contrasts between quiet characters of gibbon and noisy character of monkey" |
| Hunan | Changsha | S | 1747 | | 1 | "hou, yuan" |
| Hunan | Chenzhou | S | 1883 | | 1 | "hou, yuan"; numerous 19th century records (1802, 1815, 1817, 1859, 1866, 1867) also report "hou, yuan"; record from 1763 explicit reports "changbiyuan" |
| Hunan | Hengyang | S | 1875 | | 1 | "yuan" |
| Hunan | Huaihua | S | 1902 | | 1 | "hou, yuan" |
| Hunan | Loudi | S | 1832 | | 1 | "monkey and two types of ape"; later record from 1872 only mentions monkeys |
| Hunan | Xiangtan | S | 1756 | | 1 | "hou, yuan"; record from 1747 explicitly names "changbiyuan" |

| | | | | | | |
|---|---|---|---|---|---|---|
| Hunan | Xiangxi | S | 1930 | | 1 | "hou, yuan"; 1871 record provides description of gibbon ("long arms, good at singing") |
| Hunan | Yongzhou | S | 1901 | | 1 | "hou, yuan"; similar record from 1870; later record from 1931 only reports one type of primate called "yuan", unlike earlier reports that name both "yuan" and "hou", and so may be name transference from gibbon to monkey |
| Hunan | Yueyang | S | 1874 | | 1 | "yuan, hou"; later record from 1880 contains a few gibbon "facts" but is mostly nonsense; record from 1747 explicitly names "changbiyuan" |
| Hunan | Zhangjiajie | S | 1869 | | 1 | "like monkey but bigger, with white/dark/yellow different colour types, long arms good for climbing … good at singing, each call has three notes … can make flutes from its arm bones" |
| Hunan | Zhuzhou | S | 1763 | | 1 | "changbiyuan" |
| Jiangsu | Suzhou | S | 265-420 | 326 | 1 | |
| Jiangxi | Fuzhou | S | 1871 | | 1 | "yuan, hou" |
| Jiangxi | Ganzhou | S | 1873 | | 1 | "hou, yuan"; records up to 1873 mention both "hou" and "yuan", whereas records from 1875 only mention "hou", suggesting that "yuan" has become locally extinct |
| Jiangxi | Ji'an | S | 1087 | | 2 | |
| Jiangxi | Jingdezhen | S | 1870 | | 1 | "hou, yuan" |
| Jiangxi | Jiujiang | S | 1870 | | 1 | "hou, yuan" |
| Jiangxi | Nanchang | S | 1873 | | 1 | "hou, yuan" |
| Jiangxi | Pingxiang | S | 1860 | | 1 | "changbiyuan" |
| Jiangxi | Shangrao | S | 1872 | | 1 | "yuan"; 1873 record provides description that is not convincing - refers to its arms specifically, but otherwise only discusses its virtues/kindness, the duration of its pregnancy, and mentions that it has a short tail |
| Jiangxi | Yichun | S | 1940 | | 1 | "yuan hou"; 1873 record specifically mentions "changbiyuan", and 1860 record lists a number of primates including "yuan, hou, white yuan, golden-haired yuan" |
| Shaanxi | Ankang | N | 1731 | | 1 | "yuan (comparatively many different kinds)" |
| Shaanxi | Hanzhong | N | 1522-1566 | 1542 | 1 | "yuan, hou and small monkey" |
| Shaanxi | Shangluo | N | 1735-1796 | 1763 | 1 | Undated (late) report states that yuan, tiger and sika deer are all now locally extinct, but during the reign of the Qianlong Emperor (1735-1796) the forests were full of wild beasts, indicating that these species were regionally present during that time |

| | | | | | | |
|---|---|---|---|---|---|---|
| Shaanxi | Weinan | N | 1735 | | 1 | "at the top of the most striking, highest cliffs, the fuyuan sits squatting on the edge" |
| Shaanxi | Xi'an | N | 1779 | | 1 | "macaque, yuan [yuan female is plain/drab, and the male is black]" |
| Shandong | Linyi | N | 1736 | | 1 | "drab-coloured yuanyuan, which sadly yells tortuous cries" at Pingyi County, eastern Mengshan |
| Shanxi | Jincheng | N | 1874 | | 1 | people catch and eat yuan nao in the mountains of Yangcheng County; record also including folklore and older traditions |
| Shanxi | Linfen | N | 1760 | | 1 | at Hongdong County |
| Shanxi | Yuncheng | N | 1659 | | 1 | at Jiang County |
| Sichuan | Dazhou | N | 1932 | | 1 | "yuan, hou, small monkey" |
| Sichuan | Guangyuan | N | 762 | | 2 | at Guangyuan |
| Sichuan | Leshan | N | 1522-1566 | 1540 | 1 | "changbiyuan" |
| Sichuan | Liangshan | N | 1893 | | 1 | "yuan, hou"; also 1874 record |
| Sichuan | Luzhou | S | 8th century | 719 | 2 | at Luxian |
| Sichuan | Meishan | N | 1522-1566 | 1550 | 1 | "has macaques, has yuan" |
| Sichuan | Mianyang | N | 978 | | 2 | |
| Sichuan | Ngawa/Qiang | N | 11th century | 1073 | 2 | |
| Sichuan | Panzhihua | N | 1859 | | 1 | "changbiyuan" |
| Sichuan | Ya'an | N | 1799 | | 1 | 1941 record lists "yuanhou" but seems to refer to a single type of animal, and only lists locations where this animal is found rather than offering any description; 1931 record lists yuan, hou, nao - but only difference given between yuan and hou is that yuan has short fur whereas hou has long fur, so very unlikely to actually refer to gibbon |
| Sichuan | Yibin | S | 1522-1566 | 1552 | 1 | "yuan" [not listed together with other primates] |
| Yunnan | Baoshan | S | extant | | 6 | |
| Yunnan | Chuxiong | S | extant | | 6 | |
| Yunnan | Dali | S | 1902 | | 1 | "yuan, antelope, white yuan"; similar record from 1890 |
| Yunnan | Dehong | S | extant | | 6 | |
| Yunnan | Honghe | S | extant | | 6 | |
| Yunnan | Kunming | S | 1696 | | 1 | "yuan, hou" |
| Yunnan | Lincang | S | extant | | 6 | |

| | | | | | | |
|---|---|---|---|---|---|---|
| Yunnan | Nujiang | S | 1985 | | 5 | |
| Yunnan | Pu'er (Simao) | S | extant | | 6 | |
| Yunnan | Qujing | S | 1904 | | 1 | "changbiyuan"; later report from 1934 lists "yuan, hou", but provides very poor description that is unlikely to represent a gibbon; report from 1885 lists "yuan, hou" |
| Yunnan | Wenshan | S | 1932 | | 1 | "yuan, hou" |
| Yunnan | Xishuangbanna | S | extant | | 6 | |
| Yunnan | Yuxi | S | extant | | 6 | |
| Yunnan | Zhaotong | S | 1942 | | 1 | "yuan, hou" |
| Zhejiang | Hangzhou | S | 814 | | 2 | at Lingshandong and Moshanyudong |
| Zhejiang | Huzhou | S | 1750 | | 1 | under "monkey" category, records description of gibbon (long arms, good at singing) from Anjizhou County, Tianmushan area (including Anji and Linan in the mountains) |
| Zhejiang | Jiaxing | S | 1922 | | 1 | "yuan" in Haining |
| Zhejiang | Jinhua | S | 1753 | | 1 | "yuan, hou" in Xuanping County [= Wuxi County] |
| Zhejiang | Lishui | S | 1896 | | 1 | "yuan, hou" in Suichang; 1876 record from Jinyun reports that nowadays only monkeys and macaques occur at this locality, but in the Ming Dynasty there were gibbons as well |
| Zhejiang | Ningbo | S | 1926 | | 1 | at Chicheng [= Ninghai]; also 1927 record from Xiangshan County of gibbons that probably refers instead to macaques; also many earlier records (1223, 1792, 1879, 1888, 1899) |
| Zhejiang | Quzhou | S | 1926 | | 1 | "yuan, hou" at Qujiang; many earlier records (1701, 1711, 1822, 1873) |
| Zhejiang | Shaoxing | S | 1936 | | 1 | "yuan, hou" at Yunji [=Yue Cheng]; many earlier records (1214, 1573-1619, 1671, 1792, 1870, 1891, 1918); by 1918 described as "seldom found"; earlier records refer to both black and white gibbons |
| Zhejiang | Taizhou | S | 1934 | | 1 | "yuan, hou" at Linhai; many earlier records (1223, 1522-1566, 1894, 1926) |
| Zhejiang | Wenzhou | S | 1912 | | 1 | monkeys and different-coloured gibbons [males and females?] at Yueqing; older records (1865, 1878) list gibbons and two other types of primates |

**References:**


[1] Wen R. 2009 *The distributions and changes of rare wild animals in China*. Chongqing: Chongqing Science and Technology Press.
[2] Van Gulik RH. 1967 *The gibbon in China: an essay in Chinese animal lore.* Leiden: EJ Brill.
[3] Tan B. 1985 The status of primates in China. *Primate Conservation* **5**, 63-81.
[4] Zhou J, Wei F, Li M, Zhang J, Wang D, Pan R. 2005 Hainan black-crested gibbon is headed for extinction. *Int. J. Primatol*. **26**, 453-465.
[5] Fan P, Xiao W, Huo S, Ai H, Wang T, Lin R. 2011 Distribution and conservation status of the Vulnerable eastern hoolock gibbon *Hoolock leuconedys* in China. *Oryx* **45**, 129-134.
[6] IUCN. 2014 *The IUCN Red List of Threatened Species. Version 2014.2.* <http://www.iucnredlist.org>
