# Supplementary material for: Historical data as a baseline for conservation: reconstructing long-term faunal extinction dynamics in Late Imperial–modern China
Source: Proc Biol Sci. 2015 Aug 22;282(1813):20151299. doi: 10.1098/rspb.2015.1299 (PMC4632630; doi:10.1098/rspb.2015.1299)
Supplement: Table S4 [file rspb20151299supp4.pdf]

Table S4. Model comparison statistics. The best-fit model for each time series was selected based on a threshold of 4 ΔAIC, and is indicated by *. The simplest model was chosen as best-fit in cases where the difference in AIC across models was <4. It was not possible to fit the exponential model to the first decline section due to incompatibility with the raw data.

Decline section	Model type	AICc/AIC	ΔAIC	AIC type
1600-1850	Linear *	46.974	0	AICc
1600-1850	Quadratic	71.102	24.128	AICc
1600-1850	Exponential	NA	NA	NA
1850-2000	Linear *	32.549	1.838	AIC
1850-2000	Quadratic	30.711	0	AIC
1850-2000	Exponential	31.351	0.640	AIC
